# The Behavior Avoidance Test: Association With Symptom Severity and Treatment Outcome in Obsessive-Compulsive Disorder

**DOI:** 10.3389/fpsyt.2021.781972

**Published:** 2021-12-21

**Authors:** Jana Hansmeier, Anke Haberkamp, Julia A. Glombiewski, Cornelia Exner

**Affiliations:** ^1^Department of Psychology, University of Leipzig, Leipzig, Germany; ^2^Department of Psychology, University of Marburg, Marburg, Germany; ^3^Department of Psychology, University of Koblenz-Landau, Koblenz-Landau, Germany

**Keywords:** obsessive-compulsive disorder, Behavioral Avoidance Test (BAT), metacognitive therapy, exposure and response prevention, mechanisms of change

## Abstract

Behavior therapy of obsessive-compulsive disorder (OCD) aims to reduce avoidance, rituals, and discomfort in OCD-relevant situations. The Behavioral Avoidance Test (BAT) measures these behavior-related outcomes in individually challenging OCD-related situations. The association of the BAT with OCD severity measures and its relevance for treatment outcome is, however, still unclear. The current study investigates with a retrospective analysis of a subsample of a pilot study, (1) if reactions on the BAT are related to OCD severity measures in an OCD sample (*n* = 28), (2) if treatment with two variants of cognitive-behavior therapy (exposure and response prevention vs. metacognitive therapy) changes the BAT scores and (3) if these changes as well as pretreatment BAT avoidance are relevant for OCD treatment outcome as measured by the Yale-Brown Obsessive Compulsive Scale (Y-BOCS). Participants rated avoidance, ritual, and discomfort in three individually challenging OCD-related situations before and after therapy. For one of these situations, BAT dimensions were rated by the therapist and an independent rater in addition to the patients' ratings. Correlational analyses found significant correlations between BAT discomfort and OCD severity measures like the Y-BOCS. A repeated measures ANOVA with pre- and posttest scores showed that all three BAT dimensions significantly decreased during both treatments. Hierarchical regression analyses (controlling for Y-BOCS pretest scores) revealed that changes in BAT discomfort as well as pretreatment BAT avoidance scores predicted the Y-BOCS posttest score. These findings suggest that the BAT is a distinct measure of behavior-related outcomes partly being relevant for OCD treatment outcome.

## Introduction

Exposure and response prevention (ERP) is a highly effective treatment for obsessive-compulsive disorder (OCD) (1). One supposed mechanism of change is the experience of habituation with a significant reduction of anxiety and discomfort levels while stopping avoidance and rituals in OCD-relevant situations (2). Whereas interviews and questionnaires cannot effectively capture these processes, the Behavioral Avoidance Test (BAT) (3) allows for the assessment of behavior-related outcomes in an ecologically valid way by measuring participants' reactions to individually challenging OCD-related situations. However, the BAT has rarely been used to investigate individual behavior-related changes during ERP as well as its predictive value for treatment outcome in OCD.

The BAT asks patients to rate avoidance, rituals and discomfort during the stepwise exposure to three OCD-relevant situations. By using individual relevant OCD situations and steps for exposure, the BAT accounts for the idiosyncratic nature of OCD. It has been shown to have good psychometric properties and treatment sensitivity (3). Only the study by Steketee et al. (3) investigated the relationship of the BAT to measures of OCD symptom severity and found moderate correlations. However, due to the different methodological approach, further investigation of the relationship of the BAT to therapist- and self-rated OCD severity seems to be necessary.

As one of the dimensions of the BAT, avoidance at pretreatment could be predictive of the treatment outcome in OCD. Wheaton et al. (4) found that higher scores of behavioral avoidance as measured by one item of the Yale-Brown Obsessive-Compulsive Scale (Y-BOCS) (5) predicted a worse treatment outcome after ERP for OCD and was also associated with lower degrees of patients' adherence to between-session assignments. By applying the BAT across multiple situations, avoidance could be measured in a more individual and thus more ecologically valid way. Thus, avoidance of the BAT at pretreatment might capture the patient's level of motivation for exposing him-/herself to challenging OCD situations, which, in turn, could predict the treatment outcome of ERP. The BAT avoidance scores might thereby measure aspects of symptom severity, but additionally motivation due to its different methodological approach. This would also explain why an adapted BAT with standardized measurement of contamination-related OCD symptoms shows only moderate correlations with other measures of OCD symptom severity (6). In line with the finding by Wheaton et al. (4), BAT avoidance at pretreatment might also affect patient's compliance in exposures and behavioral experiments during treatment sessions and homework assignments, which might further affect the treatment outcome in OCD.

Cottraux et al. (7) found that BAT avoidance and discomfort levels significantly improved following cognitive therapy and behavior therapy in OCD. Especially a reduction of discomfort could be explained by habituation during treatment sessions or by a changed appraisal of the danger related to critical OCD situations. Besides ERP, it is proposed that primarily treatments incorporating behavioral experiments might affect the dimensions of the BAT and thereby the treatment outcome in OCD. As one of these, the metacognitive therapy (MCT) (8) applies verbal methods and behavioral experiments to modify beliefs about rituals or about the impact of thoughts. By going without prolonged exposures, MCT might present an effective alternative, requiring less treatment time for addressing behavior-related outcomes (9, 10). Regardless of the treatment approach, the time spent on exposures or behavioral experiments may be crucial for changing the outcomes in the BAT dimensions.

The present study aimed to investigate the association of an individually challenging BAT with OCD severity measures and its relevance for treatment outcome. Specifically, the following hypotheses were examined: 1) BAT scores are related to measures of OCD symptom severity in an OCD sample. 2) Higher avoidance of the BAT at pretreatment is predictive of a worse treatment outcome of both ERP and MCT. 3) Higher avoidance of the BAT at pretreatment is related to lower compliance in exposures and behavioral experiments during treatment sessions and homework assignments. 4) ERP and MCT can both reduce behavior-related outcomes in OCD. 5) These changes are relevant for treatment outcome in OCD as measured with global OCD symptom scores at posttreatment and follow-up. 6) The time spent with exposures/behavioral experiments during ERP/MCT is related to changes in behavior-related outcomes. In contrast to the original BAT, the present study also considered ratings by therapists and an independent rater. Considering the discrepancies between patients' and therapists' ratings reported in previous studies [e.g., (11)] these ratings might contribute to a more valid measurement, for instance by capturing rituals or avoidance behavior that patients themselves do not notice.

## Methods

### Participants

The sample consisted of 28 German-speaking individuals with the main diagnosis of OCD according to DSM-IV. They represent a subsample of the intent to treat-sample (*n* = 37) of a pilot trial comparing ERP and MCT (10). Nine patients of the intent to treat-sample did not rate the BAT so that they were not considered in the current analyses. In the analyses of changes in BAT scores from pre- to posttreatment (and their relevance for treatment outcome), only patients with complete data of the BAT at pre- and posttreatment (*n* = 19) were considered. Inclusion criteria were: (a) a diagnosis of OCD according to DSM-IV, and (b) an age of 18–65 years. Exclusion criteria were: (a) a lifetime diagnosis of substance dependence, psychosis, or neurological conditions, and (b) intellectual disability. The German version (12) of the Structured Clinical Interview (SCID) was used to assess for DSM–IV–TR current and lifetime disorders.

### Procedures

The data of the current study is part of a pilot trial comparing ERP and MCT (10), which was registered with ClinicalTrials.gov (NCT01483339). The study was approved by the Institutional Review Boards of the University of Marburg and the University of Leipzig. Participants were recruited from consecutive referrals to the universities' outpatient clinics. After screening of eligibility and informed consent, patients were randomly assigned to ERP or MCT. Random assignment was stratified by a diagnosis of comorbid depression. During the follow-up period of three months, three short telephone booster sessions following a fixed protocol took place.

### Treatment Conditions

The MCT protocol was slightly adjusted for the study (8). The original treatment schedule of ten treatment sessions was extended to 14 sessions to allow for adaptions to individual needs of patients. Verbal methods (e.g., socratic questioning about evidence, reframing advantages), detached mindfulness and behavioral experiments (e.g., ritual postponement) were applied during MCT in order to change metacognitions. According to the ERP protocol (13), prolonged ERPs were implemented in therapist-guided in-sessions and between-sessions self-exposures after preparing and planning the individual treatment (e.g., psychoeducation about habituation, hierarchy of anxiety-provoking situations). An overview of the contents of both treatment protocols is presented in Glombiewski et al. (10). Both conditions offered 14 individual weekly sessions. In ERP, one session could last longer than 50 min depending on the individual length of exposure. Thereby, the number of treatment hours (á 50 min) was significantly higher in the ERP than in the MCT condition (see Table 1).

**Table 1:** Demographic and clinical characteristics of participants.

**Variable^a^**	**Pretest (*n* = 28)**	**ERP (*n* = 8)**	**MCT (*n* = 11)**	**Statistic**	***p^b^***
**Demographics**					
Age, y	30.9 ± 10.4	25.4 ± 6.3	29.3 ± 5.9	*t*_(17)_ = −1.389	0.183
Education^c^, y	14.7 ± 2.9	15.9 ± 4.0	14.2 ± 2.2	*t*_(16)_ = 1.158	0.264
Gender, no. (%) female	22 (79)	7 (88)	8 (73)	$\chi_{\left(1\right)}^{2}$ = 0.608	0.435
**Clinical variables**					
Duration of disorder, y	6.4 ± 4.3	5.3 ± 3.9	6.5 ± 2.9	*t*_(16)_ = −0.740	0.470
Any current co-morbid disorder^d^, no. (%)	14 (50)	3 (38)	6 (55)	$\chi_{\left(1\right)}^{2}$ = 0.540	0.463
Current depression^d^, no. (%)	10 (36)	1 (13)	4 (36)	$\chi_{\left(1\right)}^{2}$ = 1.360	0.243
Y-BOCS, total, pre	23.8 ± 6.2	23.5 ± 5.6	21.1 ± 9.0	*t*_(17)_ = −0.290	0.775
BDI-II, total, pre	19.1 ± 10.4	21.1 ± 18.4	18.4 ± 12.1	*t*_(16)_ = 0.519	0.611
**BAT**					
BAT avoidance, pre	0.9 ± 0.6	0.9 ± 0.6	0.9 ± 0.6	*t*_(17)_ = −0.228	0.822
BAT rituals, pre	0.6 ± 0.5	0.7 ± 0.6	0.3 ± 0.4	*t*_(17)_ = 1.482	0.157
BAT discomfort, pre	44.8 ± 19.9	50.0 ± 16.3	44.7 ± 21.0	*t*_(17)_ = 0.593	0.561
**Treatment**					
Treatment sessions, no.	13.1 ± 1.5	12.9 ± 1.8	13.4 ± 1.3	*t*_(17)_ = −0.691	0.499
Treatment hours^e^, no.	18.0 ± 6.8	23.6 ± 6.0	13.6 ± 1.2	*t*_(7.41)_ = 4.684	**0.002**
Time exposures/behavior experiment, minutes	47.8 ± 42.8	79.9 ± 22.7	26.3 ± 40.3	*t*_(17)_ = 3.377	**0.004**

*ERP, Exposure with response prevention; MCT, Metacognitive therapy; y, years; Y-BOCS, Yale-Brown Obsessive-Compulsive Scale; BDI-II, Beck Depression Inventory-II; BAT, Behavioral Avoidance Test; BAT scores were calculated by averaging the ratings of the steps across all three situations (for the patients' ratings) and across all three raters (for the first situation)*.

a *Table values are given as mean ± SD unless indicated otherwise*.

b *Bold values indicate p < 0.05*.

c *Number of years spent in full-time education*.

d *Co-morbid mental disorder according to SCID and DSM-IV criteria (apart from OCD)*.

e *Treatment hours of 50 min*.

Both treatments were delivered by 11 doctoral-level clinical psychologists with advanced training in cognitive-behavioral therapy. All therapists were trained in both manuals and received monthly group or individual clinical supervision.

### Measures

#### Behavioral Avoidance Test

The Behavioral Avoidance Test (BAT) developed by Steketee et al. (3) is a behavior-related measure of avoidance, rituals and discomfort in OCD-critical situations of patients. In this study, the dimensions of the BAT were measured at pre- and posttreatment. The individual BAT tasks were chosen after three to four sessions of information gathering and diagnostics before treatment to enable the therapist and patient to identify all relevant OCD symptoms and triggers. According to a standardized instruction [cf., (3)], the therapist and patient jointly selected three OCD-related situations that were challenging for the patient and provoked significant discomfort, avoidance and/or rituals. Seven relevant steps, with increasing levels of difficulty for each task, were defined. Patients were informed that the BATs were a measurement of their ability to approach their feared OCD situations as far as they could proceed comfortably without ritualizing. During implementation of the BAT, the relevant steps were performed in separate attempts and ratings were performed during the procedure. One of the three tasks was not only rated by the patient, but also by the therapist and an independent rater. The independent rater was a student assistant, who was trained in the BAT and who was blind to treatment condition. This task, which was rated by all three raters, took place at the respective outpatient clinic. The other two tasks were rated by the patient only in relevant situations of daily life (e.g., at their home). After the first task was performed by all three raters at the outpatient clinic, patients were given detailed instruction how to implement the other two tasks in their daily life.

Avoidance and rituals for each step of each task were rated on a 3-point scale (from 0 = no avoidance/rituals to 2 = complete avoidance/extensive rituals). Levels of discomfort were rated on a 100-point scale of subjective units of discomfort (0 = none to 100 = extreme). For the first task, the intraclass coefficients for the subscores avoidance (*r* = 0.950, *p* < 0.001), rituals (*r* = 0.962, *p* < 0.001) and discomfort (*r* = 0.860, *p* < 0.001) at pretest indicated a good interrater reliability of the ratings by the patient, therapist, and independent rater. This allowed for the calculation of average scores for each of the three dimensions (avoidance, rituals, and discomfort) across all three raters for the first situation. In a second step, average scores for each of the dimensions were calculated by averaging the rater-combined score of the first situation with the patient-rated scores of the other two situations. The internal consistency for the scales were acceptable to good for the subscores of avoidance (Cronbach's alpha = 0.877), rituals (Cronbach's alpha = 0.853), and discomfort (Cronbach's alpha = 0.736). Thus, based on the good interrater reliability and the high internal consistency, we felt justified to calculate three overall scores for avoidance, rituals, and discomfort that were averaged across the three rater perspectives and the three situations.

Examples of the situations and related steps of the BAT are shown in the Supplementary Table 1. In addition, based on Steketee et al. (3), a global patient's composite score comprising only the patients' ratings was calculated by summing the percentage of steps, avoidance, rituals, and discomfort after dividing each variable by its standard deviation (of the whole sample). This composite score was calculated to capture in a single measure some of the various ways that performance might reflect OCD symptom severity (e.g., high avoidance of steps vs. attempts of most of the steps resulting in high levels of discomfort and/or rituals). The percentage of steps was calculated by dividing the number of completed steps by the 7 steps and multiplying with 100. The results of the analyses of the BAT composite score are shown in the Supplementary Tables 2–5.

#### Other Measures

The Yale-Brown Obsessive-Compulsive Scale (Y-BOCS) (5) is a 10-item, semi-structured, clinician-rating interview. Y-BOCS interviews and ratings were conducted by the therapist at pretreatment, posttreatment, and follow-up. The interraterreliability with an independent rater was very high (*r* = 0.93 to 0.99) (10). In addition, OCD symptom severity was measured at pretreatment with the German Padua Inventory-Palatine Revision (PI-PR) (14), a 24-item self-rating questionnaire. The compliance of patients in exposures (ERP) and behavioral experiments (MCT) during treatment sessions and homework assignments was rated by therapists each treatment session and averaged across all treatment sessions. It was rated on 6-point scale from “The patient did not attempt the assigned exposure/experiment/homework” to “The patient did more of the assigned exposure/experiment/homework than was requested” as used in the study by Primakoff et al. (15).

As the time spent on exposures and behavioral experiments might affect BAT outcomes after therapy, a variable measuring the minutes spent on exposures or behavioral experiments during the treatment sessions was considered in the analyses. It was documented by therapists after each treatment session and averaged across all treatment sessions. As can be seen in Table 1, the time spent on exposures/behavioral experiments was significantly higher in the ERP than in the MCT condition.

### Statistical Analyses

To investigate the relationship of the three BAT variables with OCD severity measures at pretreatment, Pearson correlation analyses were calculated between the respective BAT variables and the Y-BOCS as well as PI-PR scores at pretreatment. Due to a non-normally distributed variable of BAT rituals, correlations by Spearman's ρ were calculated for this variable. This hypothesis was investigated by two analyses for every BAT variable leading to an increased risk of familywise error rate. Thereby, these results are reported by applying an adjusted alpha level after Bonferroni correction (α = 0.025). The predictive value of the pretreatment BAT avoidance for treatment outcome was examined by regression analysis with the pretreatment BAT avoidance score as the independent variable (IV) and the Y-BOCS posttreatment score as the dependent variable (DV). Pretreatment scores of the Y-BOCS were entered in a first step in this regression analysis. The relationship between the pretreatment BAT avoidance score and compliance of patients in exposures/behavior experiments during treatment sessions and homework assignments was investigated by calculating Spearman's ρ between these variables, due to a non-normally distributed variable of compliance.

Changes from pre- to posttreatment and differences between treatment condition in the three BAT variables were examined by calculating separate repeated measures analyses of variances with BAT avoidance, rituals, and discomfort as the DVs. Time (pre- and posttreatment) and Group (ERP vs. MCT) were considered as IVs to investigate differences by treatment condition. The relevance of BAT scores as mechanisms of change was investigated by calculating change scores for the three BAT dimensions from pre- to posttreatment. These change scores were then entered in a regression analysis with the respective BAT change score as IV and the Y-BOCS posttreatment score and follow-up score, respectively, as the DV. Pretreatment scores of the Y-BOCS were entered in a first step in the regression analyses. To investigate the relationship between changes from pre- to posttreatment and time spent on exposures/behavior experiments during treatment sessions, correlation analyses were calculated between the respective BAT variables and the variable of time spent on exposures/behavior experiments. Due to a non-normally distributed variable of time, correlations by Spearman's ρ were calculated.

## Results

### Relationship of BAT Scores With OCD Severity Measures

The BAT avoidance scores as well as the BAT rituals scores were not significantly correlated with OCD severity measures of Y-BOCS (*r* = −0.04, *p* = 0.836 and Spearman's ρ = 0.29, *p* = 0.150) and PI-PR (*r* = 0.08, *p* = 0.707 and Spearman's ρ = 0.33, *p* = 0.104). The BAT discomfort scores were significantly correlated with the OCD severity measure of Y-BOCS (*r* = 0.39, *p* = 0.045), but not with the measure of PI-PR (*r* = 0.32, *p* = 0.118). By applying the adjusted alpha level after Bonferroni correction (α = 0.025), also the correlation of BAT discomfort and the Y-BOCS was not significant.

### Relationship of BAT Pretreatment Avoidance Scores With Treatment Outcome and Compliance

After entering Y-BOCS pretreatment score in step 1 in the regression analyses, the additional block of BAT pretreatment scores of avoidance was predictive of Y-BOCS posttreatment outcome (Δ*r*^2^ = 0.17, *p* = 0.028) as well as Y-BOCS follow-up outcome on trend level (Δ*r*^2^ = 0.11, *p* = 0.097), with higher BAT avoidance being related to higher Y-BOCS posttreatment/follow-up outcome. In addition, BAT pretreatment scores of avoidance were related to the compliance during exposures/behavior experiments on trend level (Spearman's ρ = −0.352, *p* = 0.066).

### Change in BAT Scores From Pre- to Posttreatment and Its Prediction of Treatment Outcome

**Figure 1:**
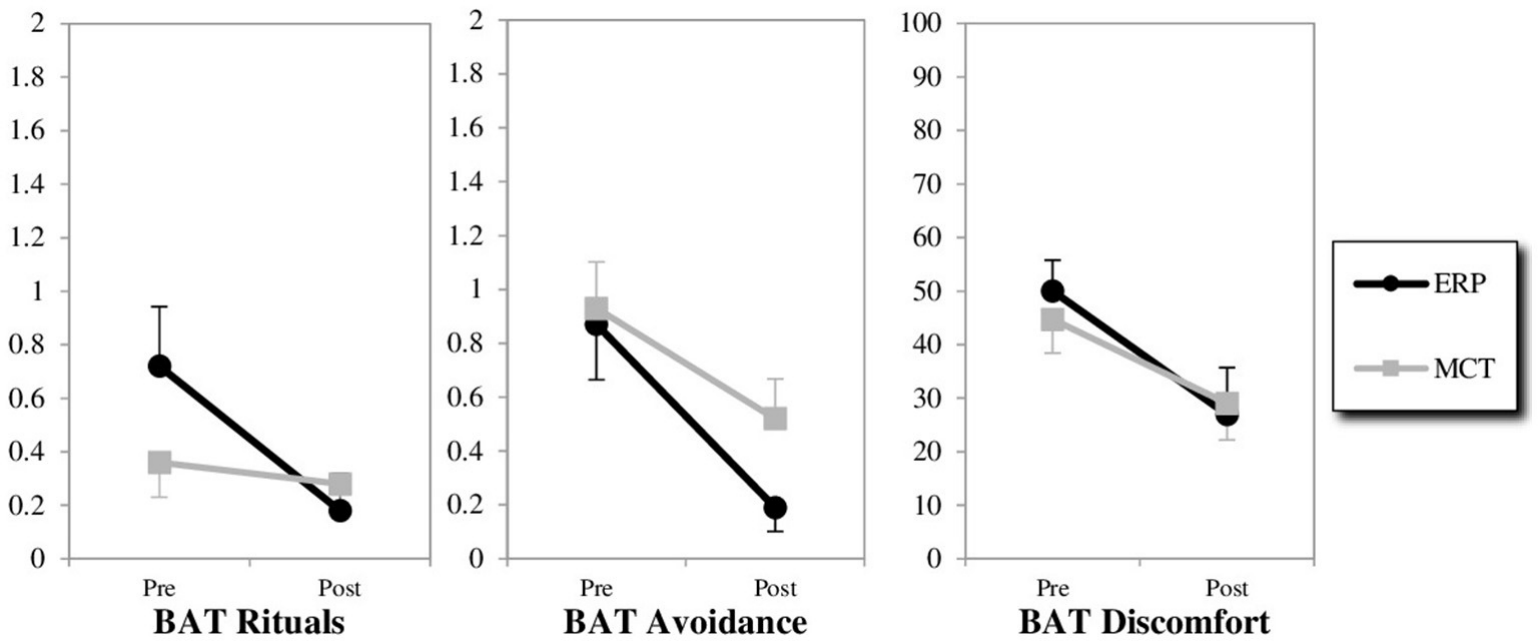
Changes from pre-to post-treatment on the behavioral avoidance test (BAT). ERP, exposure with response prevention; MCT, metacognitive therapy; BAT scores were calculated by averaging the ratings of the steps across all three situations (with a rater-combined score for the first situation).

Changes in BAT scores are displayed in Figure 1. The analyses of variances revealed that both treatments led to a significant change from pre- to posttreatment in all three BAT scores. Additionally, there was a significant effect of the group x time interaction for the outcome variable of the BAT score of rituals, with the ERP treatment condition showing greater effectiveness in decreasing rituals than MCT. For the BAT variables of avoidance and discomfort, the group x time interaction was not significant, indicating that there was no significant difference between treatments in reducing these dimensions of experience in the challenging situations (Table 2). Effect sizes for repeated measures were large for the ERP treatment condition (Cohen's d = 0.931–1.124) and small to medium for the MCT treatment condition (Cohen's d = 0.189–0.763).

**Table 2:** Statistics of the repeated measure analyses with the within-subject- factor Time (pre- and posttreatment) and the between-subject-factor Group (ERP vs. MCT).

		***F***	***df***	***P^a^***	*** ${\text{η}}_{p}^{\text{2}}$ ***
BAT avoidance	Time	15.25	17	**0.001**	0.473
	Time × Group	0.96	17	0.341	0.053
BAT rituals	Time	8.76	17	**0.009**	0.340
	Time × Group	4.79	17	**0.043**	0.220
BAT discomfort	Time	14.99	17	**0.001**	0.469
	Time × Group	0.55	17	0.470	0.031

*BAT, Behavioral Avoidance Test; BAT scores were calculated by averaging the ratings of the steps across all three situations (with a rater-combined score for the first situation)*.

a *Bold values indicate p < 0.05*.

In the regression analyses, the Y-BOCS pretreatment score was entered in step 1 of every regression analysis. The additional block of changes in BAT levels of discomfort from pre- to posttreatment was significant in predicting Y-BOCS posttreatment outcome (Δ*r*^2^ = 0.37, *p* = 0.003) as well as Y-BOCS follow-up outcome (Δ*r*^2^ = 0.26, *p* = 0.018), with higher changes in BAT discomfort being related to lower Y-BOCS posttreatment/follow-up outcome. There was a trend for the additional block of the changes in BAT rituals from pre- to posttreatment in predicting Y-BOCS posttreatment outcome (Δ*r*^2^ = 0.18, *p* = 0.060), with higher changes in BAT rituals being related to lower Y-BOCS posttreatment outcome. The results of the final model for the separate regression analyses with the respective BAT change scores from pre- to posttreatment as IVs and Y-BOCS posttreatment scores as DV are shown in Table 3.

**Table 3:** Summary statistics for the final model of the equation in the regression of the posttest Y-BOCS Score.

**Variable**	**Multiple R**	***Adj r^2^***	**Beta**	***t***	***p^a^***
**Prediction of post-treatment Y-BOCS Score by change scores BAT avoidance**					
	0.46	0.208			
Y-BOCS, pre			0.24	1.02	0.325
BAT, change score			−0.31	−1.31	0.208
**BAT rituals**					
	0.56	0.221			
Y-BOCS, pre			0.35	1.68	0.112
BAT, change score			−0.41	−2.02	0.060
**BAT Discomfort**					
	0.71	0.437			
Y-BOCS, pre			0.36	2.05	0.057
BAT, change score			−0.61	−3.44	**0.003**
**Prediction of post-treatment Y-BOCS score by pretreatment scores BAT avoidance** ^b^					
	0.62	0.326			
Y-BOCS, pre			0.59	3.23	**0.004**
BAT, pre			0.43	2.37	**0.028**

*BAT, Behavioral Avoidance Test; BAT scores were calculated by averaging the ratings of the steps across all three situations (with a rater-combined score for the first situation)*.

a *Bold values indicate p < 0.05*.

b *Due to pairwise missings, the sample size in this regression analysis was n = 23*.

### Relationship of Change Scores With Time for Exposures/Behavior Experiments

The time spent on exposures/behavior experiments was not significantly correlated with changes in BAT rituals (Spearman's ρ = 0.026, *p* = 0.915) and BAT discomfort (Spearman's ρ = 0.04, *p* = 0.875) levels. However, there was a significant positive correlation of time spent with exposures/behavior experiments and changes in BAT avoidance (Spearman's ρ = 0.64, *p* = 0.003).

Separate analyses only of the BAT scores from therapists and independent raters showed similar findings like the analyses of the combined BAT scores from all three raters. In terms of significant results, there was only one difference in the findings of analyses of variance, with a non-significant effect of the group x time interaction for the BAT score of rituals. Analyses of the BAT composite score brought similar findings like the analyses of the separate BAT dimensions, as can be seen in the Supplementary Tables 2–5.

## Discussion

This study was the first to investigate the prediction of OCD treatment outcome by an individually planned Behavioral Avoidance Test after ERP and MCT treatment. In one of three OCD-related critical situations, not only patients but also therapists and independent raters gave ratings on avoidance, rituals, and discomfort during a stepwise exposure of this situation allowing for the integration of different rater perspectives. Results showed that the BAT is a distinct measure with low relationship to measures of OCD symptom severity. Pretreatment levels of BAT avoidance significantly predicted the posttreatment outcome in OCD symptoms and were related to compliance in exposures and behavior experiments during treatment session and homework assignments. Both ERP and MCT significantly reduced all three BAT dimensions. With regard to rituals, a significant interaction effect indicated a higher decrease after ERP treatment than after MCT. Changes in BAT discomfort from pre- to posttreatment significantly predicted the outcome in OCD symptoms at posttreatment and follow-up.

A measurement of pretreatment avoidance by the BAT could reflect the motivation and willingness of patients to engage and challenge themselves during expositions and behavior experiments at the start of therapy - a factor that is difficult to assess by means of common measurements such as questionnaires and interviews. This different methodological approach might also have resulted in the low relationship between levels of BAT avoidance and measures of OCD symptom severity found at pretreatment in the current study. In line with the interpretation of a relationship with motivation, Wheaton et al. (4) found that pretreatment avoidance was also associated with the degree of patients' adherence to between-session assignments. Similarly, the current study could show that pretreatment BAT avoidance was associated with the compliance of patients in in exposures and behavioral experiments during treatment sessions and homework assignments. With regard to clinical implications, specific treatment elements such as motivation interviewing (16) may be necessary for high-avoidant patients to show the required commitment during exposures and behavioral experiments and thereby to benefit from (cognitive-) behavior therapy. Interestingly, also with regard to chronic back pain, a study (17) indicates that avoidance measured by the BAT (modified for back pain) might be an important predictor of treatment outcome in behavior therapy. Accordingly, studies [e.g., (18)] point to the necessity of applying behavioral experiments in the treatment of chronic back pain. Since behavioral marker of avoidance have also been shown to be relevant for treatment outcome in anxiety disorders [e.g., (19)], there might be a transdiagnostic relevance of this predictor for treatment outcome.

In line with previous findings (7), the present study might indicate that both ERP and MCT are not only able to improve OCD symptoms as measured by standardized interviews and questionnaires, but also in an individual Behavioral Avoidance Test with high ecological validity. The present findings give preliminary support of the relevance of a decrease of discomfort as being one important factor for treatment outcome. As one assumed mechanism of change of ERP, a reduction of discomfort might be explained by habituation during treatment sessions or by a changed appraisal of the danger related to critical OCD situations. MCT might attain these changes as well-without requiring prolonged exposures thus needing fewer treatment sessions to achieve tangible results. Only with regard to rituals, prolonged exposures as used in ERP might be especially beneficial for reducing this outcome. However, the present findings showing a significant relationship of time spent with exposures/behavior experiments and changes in BAT avoidance might indicate that, regardless of the treatment condition, a minimum amount of time spent on expositions and behavior experiments seems to be necessary to decrease avoidance during treatment. This might be an important result considering that avoidance at pretreatment was shown to be predictive of treatment outcome in both the present and in a previous study (4). Since the present study also found a relationship between pretreatment avoidance and compliance in exposures and behavior experiments, this finding might also indicate that therapists compensate for low compliance in relation to high pretreatment avoidance by spending more time for these treatment components. However, by retrospectively analyzing a subsample of a pilot study, these interpretations are still speculative and have to be verified in future studies.

One of the key advantages of the approach used in the present study is that the integration of BAT ratings from three different perspectives for one situation diminished the potential influence of factors such as lacking insight (in the case of patients) or social desirability (in the case of patients and therapists). In combination with the individual planning of three OCD-relevant situations (and their steps for exposure), this results in a global measurement with high ecological validity. Another advantage of the study is that two treatments, which clearly differ in their central treatment elements, were applied [cf., (10)]. This is important since a possible non-inferiority of MCT for affecting mechanisms that are typically attributed to ERP (reducing avoidance and discomfort) could raise a few questions, including whether (a) the MCT manual was really adhered to or else “contaminated” with elements from the ERP protocol or (b) whether the underlying mechanisms of ERP and MCT may not be that different after all. We can safely exclude that the MCT protocol was broken by exposure exercises: the high treatment fidelity we found in the pilot trial comparing ERP and MCT by Glombiewski et al. (10), suggested that MCT-specific components (such as e.g., challenging positive beliefs about rituals or about the impact of thought using verbal techniques and experiments) were delivered according to protocol without contamination. It is therefore more likely that ERP and MCT share common mechanisms that contribute to positive treatment outcomes, e.g., being able to reduce rituals and avoidance and to decrease discomfort in challenging OCD situations, but achieve these motivational and behavioral changes in patients by means of different technical approaches.

One major limitation of the study was the small sample size. The retrospective analysis of a subsample from a pilot study further limits possible conclusions about mechanisms of change and clinical implications so that the current findings only represent a start yet to be confirmed with a fully powered trial. Additionally, only one OCD-critical situation of the BAT was rated by all three raters and, since the critical BAT situations were not standardized, their difficulty levels might have varied between patients. However, the implementation of the BAT in two situations in the patients' homes allowed for BAT ratings in situations with a high relevance for their daily lives. In addition, separate analyses only of the BAT scores from therapists and independent raters showed similar findings like the analyses of the combined BAT scores from all three raters. The standardized manual of the BAT resulted in a standardized procedure for defining OCD-critical situations and their steps of exposure, which was also applied to the two situations that the patients rated by themselves at home.

The present study might have some clinical implications suggesting that the patients' willingness to expose themselves in BAT situations could be an important predictor of treatment outcome. Regardless of treatment condition, these findings might indicate that therapists should spend a minimum amount of time with exposure exercises and/or behavior experiments to reduce avoidance (what might also help to compensate for low compliance being associated with high pretreatment avoidance). MCT treatment techniques such as changing beliefs about the importance of thoughts and rituals by using verbal methods and behavioral experiments might be an economical and effective alternative to ERP for reducing levels of avoidance, rituals, and discomfort in OCD-critical situations. The findings of the present study justify a larger trial with a focus on behavior-related predictors and changes, their relevance for treatment outcome and the treatment elements affecting them.

## Data Availability Statement

The raw data supporting the conclusions of this article will be made available by the authors, without undue reservation.

## Ethics Statement

The studies involving human participants were reviewed and approved by the Institutional Review Boards of the University of Marburg and of the University of Leipzig. The patients/participants provided their written informed consent to participate in this study.

## Author Contributions

CE and JG initiated the study. CE, JG, and JH designed the study and wrote the study proposal. JH analyzed the data and drafted the manuscript. CE, JG, and AH revised the manuscript. All authors helped in collecting the data, contributed to the interpretation of data, critically reviewed for important intellectual contents, and gave the final approval of the version to be published.

## Funding

The study was supported by Ph.D. scholarships from the non-profit organization Psychotherapieambulanz Marburg e.V. (PAM e.V.) to JH and AH. We acknowledge support from Leipzig University for Open Access Publishing.

## Conflict of Interest

The authors declare that the research was conducted in the absence of any commercial or financial relationships that could be construed as a potential conflict of interest.

## Publisher's Note

All claims expressed in this article are solely those of the authors and do not necessarily represent those of their affiliated organizations, or those of the publisher, the editors and the reviewers. Any product that may be evaluated in this article, or claim that may be made by its manufacturer, is not guaranteed or endorsed by the publisher.
