# Supplementary material for: The Behavior Avoidance Test: Association With Symptom Severity and Treatment Outcome in Obsessive-Compulsive Disorder
Source: Front Psychiatry. 2021 Dec 21;12:781972. doi: 10.3389/fpsyt.2021.781972 (PMC9231550; doi:10.3389/fpsyt.2021.781972)
Supplement: Supplementary file 1 [file Data_Sheet_1.docx]

**Supplement: Examples of BAT situations**

*Table S1: Examples of situations and according steps of the Behavioral Avoidance Task (BAT)*

|  |  | Steps of the BAT^a^ | | | | | | |
| --- | --- | --- | --- | --- | --- | --- | --- | --- |
| **Subtype** | Situa-tion | 1 | 2 | 3 | 4 | 5 | 6 | 7 |
| **Washing compulsion** | Public toilet | Touching toilet seat during daylight | Touching toilet seat in the dark | Touching flush with not-inflamed fingertip | Touching flush with inflamed fingertip | Touching toilet seat with not-inflamed fingertip | Touching toilet seat with inflamed fingertip | Touching toilet seat with the whole hand |
| **Checking compulsion** | Lea-ving the kitchen | Turning oven on and off | Turning oven on and off with pan on it | Step 2 plus leaving the room | Step 3 plus staying away for 1 hour | Step 4 plus staying away for 4 hours | Step 5 plus coming home without checking | Step 6 plus going to bed without checking |
| **Aggressive impulses** | Hand-ling sharp knife | Puting knife on desk | Looking at knife for 30 seconds | Looking at knife for 5 minutes | Being in the kitchen with husband and knife | Holding knife in hand in the presence of husband | Step 5 plus approach-ing husband up to 2 metres | Step 5 plus being very close to husband (touching possible) |

^a^ Mean scores of avoidance, rituals and discomfort of the BAT were calculated by averaging the ratings of the 7 steps across all three situations (with a rater-combined score for the first situation).

**Supplement: Results of the Composite Score**

| **Outcome** |  | ***r*** | ***p*** |
| --- | --- | --- | --- |
| **BAT Patient Composite Score** | Y-BOCS | .33 | .111 |
|  | PI-PR | .41 | **.049** |

*Table S2: Pearson correlation coefficients at pretrest with OCD symptom severity measures*

Y-BOCS: Yale-Brown Obsessive Compulsive Scale, PI-PR: Padua Inventory - Palatine Revision, Due to pairwise missings, the sample size was: n = 25 (Y-BOCS) and n = 24 (PI-PR); The BAT patient composite score was calculated by summing percentage of steps, avoidance, rituals and discomfort after dividing each variable by its standard deviation (of the whole sample).

| **Outcome** |  | **Pre** | | **Post** | |  |
| --- | --- | --- | --- | --- | --- | --- |
|  |  | ***M*** | ***SD*** | ***M*** | ***SD*** | **ES** |
| **BAT Patient Composite Score** | ERP | 5.87 | 0.90 | 3.91 | 1.99 | **1.083** |
|  | MCT | 5.01 | 1.15 | 4.21 | 1.11 | **0.701** |

*Table S3: Effect Sizes (d_Repeated Measures_) from Pre- to Post-Treatment (Pre-Post) for Completers of the Behavioral Avoidance Task (BAT)*

ERP: Exposure with Response Prevention, MCT: Metacognitive Therapy, sample size: n=8 ERP vs. n=9 MCT; The BAT patient composite score was calculated by summing percentage of steps, avoidance, rituals and discomfort after dividing each variable by its standard deviation (of the whole sample).

|  |  | ***F*** | ***df*** | ***p*** | ***η^2^_p_*** |
| --- | --- | --- | --- | --- | --- |
| **BAT Patient Composite Score** | Time | 14.40 | 15 | **.002** | .490 |
|  | TimexGroup | 2.55 | 15 | .131 | .145 |

*Table S4: Statistics of the repeated measure analyses with the within-subject-factor Time (pre- and posttreatment) and the between-subject-factor Group (ERP vs. MCT)*

BAT: Behavioral Avoidance Test, sample size: n=8 ERP vs. n=9 MCT; The BAT patient composite score was calculated by summing percentage of steps, avoidance, rituals and discomfort after dividing each variable by its standard deviation (of the whole sample).

| Variable | ***Multiple R*** | ***Adj r²*** | ***Beta*** | ***t*** | ***Significance*** |
| --- | --- | --- | --- | --- | --- |
| **BAT Patient Composite Score** | | | | | |
|  | .60 | .268 |  |  |  |
| YBOCS Pre |  |  | .23 | 1.06 | .306 |
| BAT Change |  |  | -.51 | -.36 | **.033** |

*Table S5: Summary statistics for the final of the equation in the regression of the Posttest YBOCS Score*

BAT: Behavioral Avoidance Test, n=17; The BAT patient composite score was calculated by summing percentage of steps, avoidance, rituals and discomfort after dividing each variable by its standard deviation (of the whole sample).
